# HBV suppresses macrophage immune responses by impairing the TCA cycle through the induction of CS/PDHC hyperacetylation

**DOI:** 10.1097/HC9.0000000000000294

**Published:** 2023-10-12

**Authors:** Jiaxin Bei, Ye Chen, Qianbing Zhang, Xiaobin Wang, Liteng Lin, Jingjun Huang, Wensou Huang, Mingyue Cai, Weiguo Cai, Yongjian Guo, Kangshun Zhu

**Affiliations:** 1 Laboratory of Interventional Radiology, Department of Minimally Invasive Interventional Radiology and Department of Radiology, The Second Affiliated Hospital, Guangzhou Medical University, Guangzhou, Guangdong Province, China; 2 Department of Radiology, The Second Affiliated Hospital, Guangzhou Medical University, Guangzhou, Guangdong Province, China; 3 Cancer Research Institute, School of Basic Medical Sciences, Southern Medical University, Guangzhou, Guangdong Province, China

## Abstract

**Background:**

It is now understood that HBV can induce innate and adaptive immune response disorders by affecting immunosuppressive macrophages, resulting in chronic HBV infection. However, the underlying mechanism is not fully understood. Dysregulated protein acetylation can reportedly influence the differentiation and functions of innate immune cells by coordinating metabolic signaling. This study aims to assess whether HBV suppresses macrophage-mediated innate immune responses by affecting protein acetylation and to elucidate the underlying mechanisms of HBV immune escape.

**Methods:**

We investigated the effect of HBV on the acetylation levels of human THP-1 macrophages and identified potential targets of acetylation that play a role in glucose metabolism. Metabolic and immune phenotypes of macrophages were analyzed using metabolomic and flow cytometry techniques. Western blot, immunoprecipitation, and immunofluorescence were performed to measure the interactions between deacetylase and acetylated targets. Chronic HBV persistent infected mice were established to evaluate the role of activating the tricarboxylic acid (TCA) cycle in macrophages for HBV clearance.

**Results:**

Citrate synthase/pyruvate dehydrogenase complex hyperacetylation in macrophages after HBV stimulation inhibited their enzymatic activities and was associated with impaired TCA cycle and M2-like polarization. HBV downregulated Sirtuin 3 (SIRT3) expression in macrophages by means of the toll-like receptor 2 (TLR2)-NF-κB- peroxisome proliferatoractivated receptor γ coactivator 1α (PGC-1α) axis, resulting in citrate synthase/pyruvate dehydrogenase complex hyperacetylation. *In vivo* administration of the TCA cycle agonist dichloroacetate inhibited macrophage M2-like polarization and effectively reduced the number of serum HBV DNA copies.

**Conclusions:**

HBV-induced citrate synthase/pyruvate dehydrogenase complex hyperacetylation negatively modulates the innate immune response by impairing the TCA cycle of macrophages. This mechanism represents a potential therapeutic target for controlling HBV infection.

## INTRODUCTION

HBV infection poses a major health burden worldwide. In some populations, host immune dysfunction during the natural course of HBV infection can result in chronic persistent infection that can lead to fibrosis, cirrhosis, and even HCC. Globally, an estimated 250 million individuals are chronically infected with HBV, and about 1 million individuals die each year from HBV-related liver disease.^1^ Current evidence suggests that the currently approved treatment (nucleos(t)ide analogs) exhibits limited effectiveness in achieving a functional cure for chronic HBV infection.^2,3^

The innate and adaptive immune response disorders have been considered a critical obstacle to HBV clearance.^4^ As important nonparenchymal cells in the liver, Kupffer cells and peripheral blood mononuclear cells (PBMC)-derived macrophages often undergo M1 immune-activated or M2 immunosuppressive polarization due to their phenotypic plasticity, thereby regulating host innate immune responses.^5^ It has been reported that HBV polarizes macrophages toward the M2 phenotype and stimulates the secretion of IL-10 and other inhibitory cytokines,^6,7^ which can facilitate HBV immune escape by means of limiting innate immunity. Additionally, it was found that the expression of IL-10 and inhibitory cell surface molecules of monocytes in patients with chronic HBV was significantly higher than in healthy individuals.^8^ Nevertheless, since the exact mechanisms have not been clarified, further investigation is needed to understand how these findings might be translated into therapeutic strategies for patients with chronic HBV infection.

Acetylation is an essential post-translational modification that regulates protein function and is ubiquitous in enzymes that catalyze intermediate metabolism.^9^ Acetylases reportedly use intermediate metabolites from the tricarboxylic acid (TCA) cycle as substrates for acetyl donors to carry out the acetylation of lysine residues in a target protein, while deacetylases are responsible for removing acetyl units from the lysine residues.^10^ It is now understood that changes in cellular metabolism can alter the effector functions of innate immune cells.^11^ Notably, two-thirds of TCA cycle enzymes are reportedly acetylated in response to pathogen challenge,^12,13^ suggesting that acetylation may play a direct role in metabolic regulation by affecting the function of modified TCA cycle enzymes and thus participate in initiating innate immune responses. For example, deacetylation of pyruvate dehydrogenase complex (PDHC) can enhance its enzymatic activity, increasing TCA cycle flux within macrophages and further activating IL-1β–related proinflammatory pathways.^14,15^ However, it remains unknown if acetylation participates in HBV-induced macrophage M2-like polarization.

In the present study, we found that HBV could induce hyperacetylation of citrate synthase (CS) and PDHC in macrophages. CS/PDHC hyperacetylation markedly inhibited their enzymatic activities, associated with impaired TCA cycle and M2-like polarization of macrophages. Mechanistically, HBV downregulated Sirtuin 3 (SIRT3) expression through activation of the toll-like receptor 2 (TLR2)-NF-κB-peroxisome proliferatoractivated receptor γ coactivator 1α (PGC-1α) axis in macrophages, thereby increasing the acetylation of CS/PDHC. By constructing a chronic HBV-infected mouse model, we provide evidence that activation of the TCA cycle can effectively prevent the M2-like polarization, reducing HBV DNA loads and delaying the progression of HBV infection to hepatic fibrosis. Our findings reveal a novel mechanism of HBV immune escape and provide further insights to develop effective therapeutic strategies for improving the clinical outcome of patients with chronic HBV infection.

## METHODS

### Collection of HBV particle

The human hepatoblastoma cell line HepG-2.2.15 (KeyGEN Biotech Co. Ltd., Jiangsu, China) was used to generate and collect HBV particles as described.^16^ Briefly, HepG-2.2.15 cells were grown in DMEM (11965092, Gibco, NY) containing 10% fetal bovine serum without antibiotic-antimycotic supplementation. When cell density reached ~70%–80% confluence, supernatants were harvested and mixed with PEG-8000 powder (10%). After 12 hours incubation with shaking at 4°C, the mixture was centrifuged at 1000*g* for 30 minutes at 4°C. The sediment samples were subsequently obtained and resuspended in serum-free Roswell Park Memorial Institute-1640 (11875093, Gibco, NY). The HBV titers were determined by real-time quantitative PCR analyses using an HBV nucleic acid detection kit (sansure001, Sansure bioTech, Changsha, China). Every 2 million cells were treated with 1 mL of culture medium containing 10^7^ IU/mL HBV at a multiplicity of infection of ~50 genome equivalents in our study. According to the Asia-Pacific guidelines, 1 IU/mL corresponds to approximately 5 copies/mL, while 1 copy corresponds to 1 genome equivalent.^17^

The human hepatoblastoma cell line HepG-2 (KeyGEN Biotech Co. Ltd., Jiangsu, China) was cultured in DMEM (11965092, Gibco, NY). Supernatants were harvested and subjected to the same pretreatment as the HepG-2.2.15 cell line to generate a conditioned medium. This conditioned medium was used as a control for *in vitro* experiments.

### Animal procedures

C57BL/6 mice (male, 4 wk old; Gempharmatech Co., Ltd., Guangdong, China) were housed and bred under a 12-hour light/dark cycle with free access to standard diet and water. All animal procedures were performed in accordance with the Reporting of *in vivo* Experiments (ARRIVE) guidelines and received ethical approval from the Laboratory Animal Ethics Committee of the Second Affiliated Hospital, Guangzhou Medical University (Approval No: A2020-045). The chronic HBV-infected mouse model was constructed by adeno-associated virus vector mediated HBV genome transduction.^18^ Mice received a single tail vein injection of 1 × 10^11^ vg/100 μL recombinant adeno-associated virus (rAAV)-1.3 HBV (FivePlus Molecular Medicine Institute, Beijing, China). Blood samples were collected weekly from the submandibular vein of all mice and tested for HBV-related serological markers (HBeAg, HBsAg, and HBV DNA). HBeAg and HBsAg were measured using an electrochemiluminescence system (Roche Diagnostics, Mannheim, Germany). Six weeks after rAAV-1.3 HBV injection, mice were treated with dichloroacetate (DCA, 100 mg/kg^19^) or an equivalent volume of saline (0.9% NaCl) once daily by means of i.p. injection, and the blood samples were collected for HBV-related serological marker detection at the indicated time points. Following 10 weeks of DCA treatment, all mice were anesthetized with isoflurane and sacrificed by cervical dislocation; the liver and spleen tissues were harvested for subsequent assays.

### Statistical analysis

Experimental data were presented as the mean± SD, and statistical analysis was carried out using GraphPad Prism 8 (GraphPad Software, CA). One-way (for multiple groups) or 2-way ANOVA (for multiple groups and factors) followed by Tukey *post hoc* testing was used for multiple group comparisons. The data for clinical patients were statistically analyzed using a paired Student *t* test. Differences were considered statistically significant when the *p*‐value was <0.05.

A comprehensive description of all methods is provided in the Supplemental Methods section, http://links.lww.com/HC9/A582.

## RESULTS

### HBV induces CS/PDHC hyperacetylation in macrophages

**FIGURE 1:**
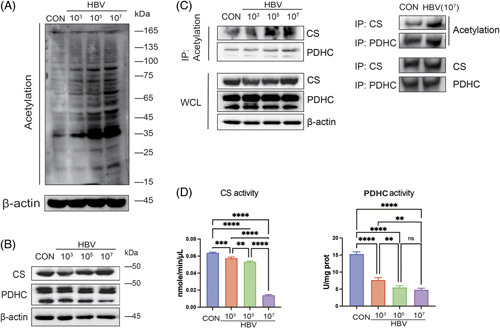
Enzymatic activities of CS and PDHC can be inhibited in macrophages by HBV-induced hyperacetylation. (A) Changes in global acetylation levels in human THP-1 macrophages infected with different HBV titers (0, 10^3^, 10^5^, 10^7^ IU/mL). Treatment condition at the HBV titers of 0 IU/mL was used as a control group (CON). β-Actin was used as a loading control. (B) Effect of HBV on CS and PDHC abundance in human THP-1 macrophages. (C) IP analysis of CS and PDHC hyperacetylation in human THP-1 macrophages following exposure to HBV (10^7^ IU/mL). (D) CS and PDHC enzymatic activities in human THP-1 macrophages following treatment with different HBV titers. Data presented as the mean ± SD, n=3, ns=*p*>0.05, **p<0.01, ***p<0.001, ****p<0.0001 (1-way ANOVA with Tukey post hoc test). Abbreviations: CS, citrate synthase; IP, immunoprecipitation; PDHC, pyruvate dehydrogenase complex; WCL, whole cell lysate.

To investigate the impact of HBV on the acetylation of macrophages, we detected the global acetylation level of human THP-1 macrophages following infection with different HBV titers (0, 10^3^, 10^5^, and 10^7^ IU/mL) for 48 hours. The global acetylation level of macrophages was strongly elevated following exposure to 10^7^ IU/mL of HBV compared to the other cultures (0, 10^3^, 10^5^ IU/mL; Figure 1A).

Given that acetylation is closely associated with the TCA cycle in cellular metabolism,^20^ we assessed the expression levels of key TCA cycle enzymes (ie, CS and PDHC) in human THP-1 macrophages. However, no significant changes were observed in the expression of CS or PDHC in macrophages following exposure to different HBV titers, suggesting that the effect of HBV on global acetylation was not caused by the altered expression of CS and PDHC (Figure 1B). Nonetheless, immunoprecipitation (IP) analysis found that CS and PDHC were modified by acetylation. The acetylation levels of CS and PDHC in macrophages were elevated with increased HBV titers (Figure 1C). It is also worth noting that exposure to 10^7^ IU/mL of HBV significantly inhibited the enzymatic activities of CS and PDHC in macrophages (Figure 1D).

Collectively, these findings suggest that the hyperacetylation of CS and PDHC induced by 10^7^ IU/mL of HBV negatively affected their enzymatic activity. This acetylation-mediated modulation might lead to impairment of the TCA cycle in macrophages.

### M2-like polarization driven by HBV is accompanied by the impairment of the TCA cycle

**FIGURE 2:**
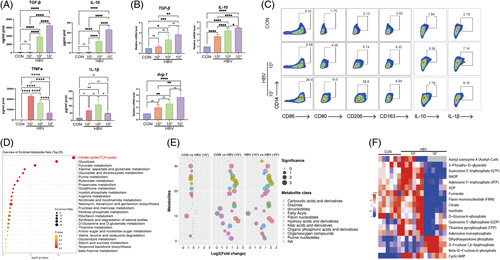
HBV stimulation drives the polarization of macrophages toward the M2 phenotype and inhibits the TCA cycle. (A) Secretion of TGF-β1, IL-10, TNFα, and IL-1β in human THP-1 macrophages following exposure to different HBV titers (0, 10^3^, 10^5^, 10^7^ IU/mL). Data presented as the mean ± SD, n=3, ns=*p*>0.05, **p<0.01, ***p<0.001, ****p<0.0001 (1-way ANOVA with Tukey post hoc test). (B) Real-time quantitative PCR analysis of changes in TGF-β1, IL-10, and Arg-1 mRNA levels in human THP-1 macrophages following exposure to different HBV titers (00, 10^3^, 10^5^, 10^7^ IU/mL). Relative mRNA levels are presented as the mean ± SD, n=6, ns=*p*>0.05, **p<0.01, ***p<0.001, ****p<0.0001 (1-way ANOVA with Tukey post hoc test). (C) After exposure to HBV (0, 10^3^, or 10^7^ IU/mL) for 48 hours, expressions of CD80, CD86, CD163, CD206, IL-10, and IL-1β were measured by flow cytometry. (D) Metabolic enrichment analysis in human THP-1 macrophages; the SMPBD database was used as a reference. (E) Targeted metabolomic data comparison among 3 groups of samples. Different metabolite classes are represented with different colors. Circle sizes reflect the significance of the log2 fold change measured. Con=0 IU/mL HBV. (F) Heatmap of differential metabolites associated with glucose metabolism in human THP-1 macrophages following exposure to different HBV titers (0, 10^3^, 10^7^ IU/mL) stimulation. Data presented as the mean ± SD, n=6. Abbreviations: FMN, flavin mononucleotide; GDP, guanosine diphosphate; GTP, guanosine triphosphate; TCA, tricarboxylic acid; TPP, thiamin pyrophosphate.

We first examined whether HBV stimulation could induce immunophenotypic polarization of macrophages. As shown in Figure 2A, besides 10^3^ IU/mL HBV, higher HBV titers (10^5^ and 10^7^ IU/mL) promoted human THP-1 macrophages secretion of anti-inflammatory cytokine (TGF-β1 and IL-10), while decreasing the production of TNFα and IL-1β. In addition, real-time quantitative PCR analysis demonstrated that the mRNA levels of *TGF-β1, IL-10*, and *Arg-1* were upregulated in human THP-1 macrophages following exposure to 10^5^ and 10^7^ IU/mL of HBV (Figure 2B). These results were further verified in human PBMC-derived macrophages. Flow cytometry results confirmed that 10^7^ IU/mL of HBV could drive the polarization of macrophages toward the immunosuppressive M2-like phenotype (Figure 2C).

Next, we used label-free mass spectrometry to quantify the glucose metabolites in human THP-1 macrophages following exposure to different HBV titers (0, 10^3^, 10^7^ IU/mL). The results showed that the commonly enriched metabolite sets detected in different samples were primarily associated with the TCA cycle, glycolysis/gluconeogenesis, and the pyruvate pathway (Figure 2D). The significantly different metabolite classes between samples included carboxylic acids and their derivatives, dinucleotides, flavin nucleotides, keto acids and their derivatives, organic phosphoric acids and their derivatives, and purine nucleotides (Figure 2E). The differences in metabolic patterns within the macrophages between higher HBV titers (10^7^ IU/mL) and lower titers (0, 10^3^ IU/mL) were visualized in heatmaps (Figure 2F). Specifically, the levels of multiple TCA cycle-related metabolites, including oxaloacetate, cis-aconitate, citrate, isocitrate, ATP, and NAD, were markedly decreased in human THP-1 macrophages following exposure to 10^7^ IU/mL of HBV compared with the other treatment conditions (0, 10^3^ IU/mL; Supplemental Figure S1, http://links.lww.com/HC9/A583).

Collectively, these findings demonstrated that HBV-mediated M2-like macrophage polarization was associated with glucose metabolism reprogramming characterized by an impaired TCA cycle.

### HBV inhibits CS/PDHC enzyme activities by downregulating SIRT3 expression in macrophages and drives polarization toward the M2-like phenotype

**FIGURE 3:**
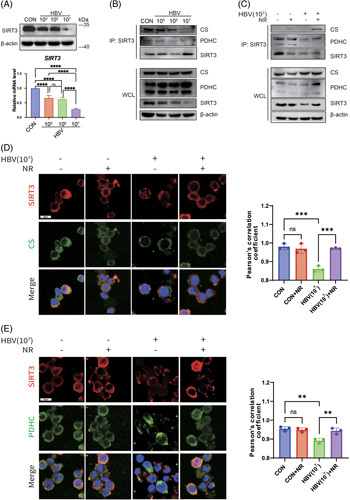
HBV induces CS and PDHC hyperacetylation by downregulating SIRT3 expression in macrophages. (A) SIRT3 expression at the protein and mRNA levels in human THP-1 macrophages following exposure to HBV. Upper: western blots analysis with β-actin as a loading control. Bottom panel: The mRNA levels are measured by real-time quantitative PCR and normalized to internal control beta-actin (ACTB). Relative mRNA levels are presented as the mean ± SD, n=6, *=*p*<0.05, **p<0.01, ****p<0.0001 (1-way ANOVA with Tukey post hoc test). (B) Co-IP analysis of the interactions between SIRT3 and CS and PDHC in human THP-1 macrophages with and without exposure to HBV. (C) The effect of NR-mediated SIRT3 activation on the interactions between SIRT3 and CS and PDHC in human THP-1 macrophages. (D and E) Colocalization of SIRT3 and CS and PDHC in human THP-1 macrophages. Left: representative confocal images of the co-localization of SIRT3 with CS (D) and PDHC (E). Nuclei are stained with DAPI (blue) (magnification: 63×, scale bar: 20 μm). Right: quantification of SIRT3 and CS/PDHC co-localization is presented as Pearson correlation coefficient. Histograms show statistical analysis of the degree of colocalization between SIRT3 and CS/PDHC based on Pearson correlation coefficients (mean ± SD). n=3, ns=*p*>0.05, **p<0.01, ***p<0.001 (1-way ANOVA with Tukey post hoc test). Abbreviations: CS, citrate synthase; IP, immunoprecipitation; NR, nicotinamide riboside; PDHC, pyruvate dehydrogenase complex; SIRT3, Sirtuin 3; WCL, whole cell lysate.

It has been established that SIRT3 is the major deacetylase in cellular mitochondria.^21^ Besides, bioinformatic analysis based on the data set obtained from the Gene Expression Omnibus database revealed that the SIRT3 expression of liver macrophages in HBV-infected mice was reduced relative to healthy mice (Supplemental Figure S2, http://links.lww.com/HC9/A584). Hence, we hypothesized that SIRT3 might contribute to the mechanism by which HBV infection induces CS and PDHC hyperacetylation in macrophages. We found that in contrast to the control (0 IU/mL HBV), the SIRT3 protein and mRNA levels in human THP-1 macrophages were downregulated following HBV (10^3^, 10^5^, 10^7^ IU/mL) stimulation, of which 10^7^ IU/mL of HBV induced the most significant downregulation (Figure 3A). Using the purified glutathione-S-transferase-tag SIRT3 protein and His-tag CS/PDHC protein (HIS-CS/PDHC), glutathione-S-transferase pull-down assay results confirmed direct binding between SIRT3 and CS and PDHC (Supplemental Figure S3, http://links.lww.com/HC9/A585), suggesting that SIRT3 interacts with CS and PDHC. The Co-IP assay validated that the interactions between SIRT3 and CS and PDHC were significantly weaker in human THP-1 macrophages after HBV (10^7^ IU/mL) stimulation (Figure 3B); however, this phenomenon could be reversed by nicotinamide riboside chloride (NR), an agonist of SIRT3 deacetylase activity.^22^ Meanwhile, NR administration yielded no apparent effect on the abundance of SIRT3 protein (Figure 3C).

Consistent with these observations, immunofluorescence analysis demonstrated that SIRT3 was significantly colocalized with CS and PDHC in human THP-1 macrophages, while HBV reduced this effect. In contrast, the administration of NR significantly increased co-localizations of SIRT3 and CS, SIRT3 and PDHC in macrophages in the presence of HBV (Figure 3D, E). These findings provide compelling evidence that HBV attenuates the interactions of SIRT3 with CS and PDHC by downregulating SIRT3 expression in macrophages, leading to CS and PDHC hyperacetylation.

**FIGURE 4:**
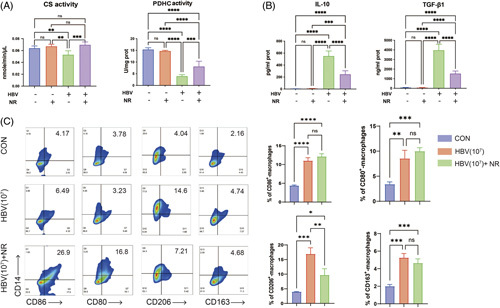
Modulation of CS and PDHC enzyme activities by SIRT3 impacts the immune response of HBV-induced macrophages. (A) Effect of enhanced SIRT3 deacetylase activity on CS and PDHC activity in human THP-1 macrophages exposed to HBV (10^7^ IU/mL). Data presented as the mean ± SD, n=3, ns=*p*>0.05, **p<0.01, ****p<0.0001 (1-way ANOVA with Tukey post hoc test). (B) Effect of NR-mediated SIRT3 activation on anti-inflammatory (TGF-β1 and IL-10) cytokine secretion in human THP-1 macrophages. Data presented as the mean ± SD, n=5, ns=*p*>0.05, **p<0.01, ***p<0.001, ****p<0.0001 (1-way ANOVA with Tukey post hoc test). (C) Expression of M1 markers (CD80, CD86) and M2 markers (CD163, CD206) in human PBMC-derived macrophages (left: Representative flow cytometric plot; right: statistical graphs). Abbreviations: CS, citrate synthase; NR, nicotinamide riboside; PDHC, pyruvate dehydrogenase complex; PBMC, peripheral blood mononuclear cell; SIRT3, Sirtuin 3.

We subsequently investigated whether enhancing SIRT3 deacetylase activity by means of NR treatment could improve CS and PDHC enzymatic activities in HBV-induced macrophages. Obviously, NR could effectively rescue the reduced CS and PDHC activities induced by 10^7^ IU/mL HBV (Figure 4A). However, no increase in CS/PDHC enzymatic activity was observed in human THP-1 macrophages following NR administration without HBV stimulation. Besides, human THP-1 macrophages treated with NR alone exhibited no significant changes in the secretion of anti-inflammatory cytokines (TGF-β1 and IL-10) compared to untreated cells. However, in the presence of 10^7^ IU/mL of HBV, NR inhibited TGF-β1 and IL-10 secretion (Figure 4B). Similar immune-activating effects of NR were observed in human PBMC-derived macrophages. As shown in Figure 4C, NR treatment led to decreased expression of M2-like macrophage surface markers (CD206) in HBV-induced macrophages.

To verify the effect of SIRT3 downregulation induced by HBV on macrophage phenotype, human THP-1 macrophages were infected with a control lentivirus (vector) or lentivirus (Flag-SIRT3) to overexpress SIRT3 (Supplemental Figure S4A, http://links.lww.com/HC9/A586). By performing IP experiments, we found that SIRT3 overexpression could prevent HBV-induced hyperacetylation of CS/PDHC in macrophages (Supplemental Figure S4B, http://links.lww.com/HC9/A586). Further, the Co-IP assay revealed that inhibition of SIRT3 binding to CS and PDHC in macrophages, induced by 10^7^ IU/mL of HBV, was significantly rescued by means of SIRT3 overexpression (Supplemental Figure S4C, http://links.lww.com/HC9/A586), which would promote CS and PDHC deacetylation. Following HBV exposure, SIRT3-overexpressing macrophages led to significantly increased proportions of CD86^+^ cells and decreased CD206^+^ cells compared to cells infected with vectors (Supplemental Figure S4D, http://links.lww.com/HC9/A586).

These results indicate that HBV downregulated SIRT3 to modulate the enzymatic activities of CS and PDHC in macrophages while contributing to the impairment of the TCA cycle. This process played an important role in inducing macrophage M2-like polarization.

### HBV downregulates SIRT3 expression in macrophages by means of the TLR2-NF-κB-PGC-1α axis

**FIGURE 5:**
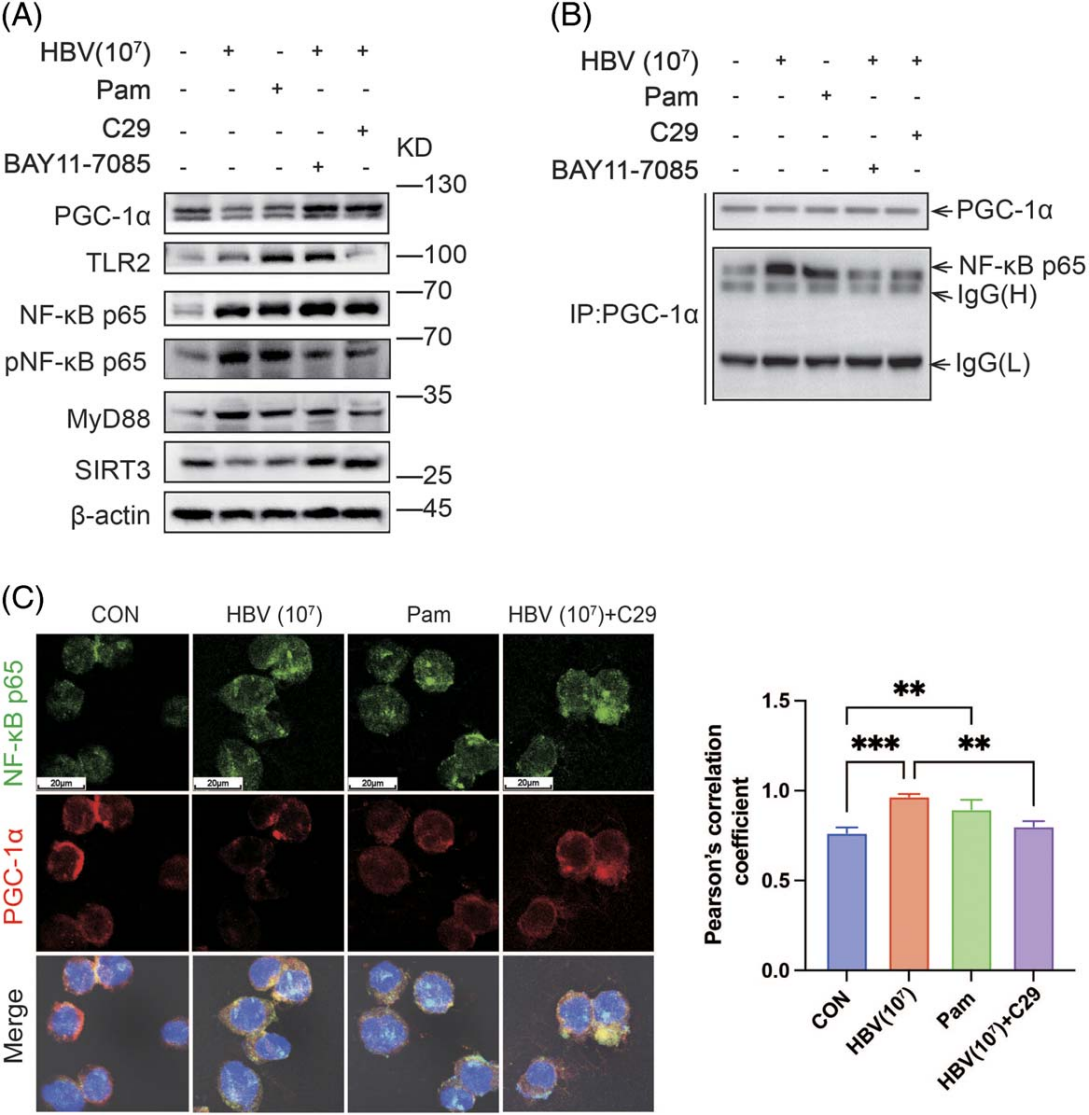
HBV stimulation regulates the expression of SIRT3 in macrophages through the TLR2-NF-κB-PGC-1α axis. (A) The inhibitory effect of HBV stimulation on SIRT3 expression was closely associated with aberrant activation of the TLR2-NF-κB-PGC-1α axis. TLR2 agonist Pam was used as a means of activating TLR2-NF-κB signaling, while TLR2 inhibitor C29 and NF-κB activation inhibitor BAY11-7085 were used to suppress TLR2-NF-κB signaling in human THP-1 macrophages. β-Actin was used as a loading control. (B) Co-IP analysis of PGC-1α binding to the p65 subunit of NF-κB, the interaction was positively regulated by HBV stimulation or Pam but inhibited by C29 or BAY11-7085. (C) Immunofluorescence evaluation of the correlation between PGC-1α/NF-κB p65 binding and activation of the TLR2-NF-κB signaling. Left panel: representative confocal images. DAPI staining (blue) indicates the nuclei (magnification: 63×, scale bar: 20 μm). Right: statistical analysis of the degree of colocalization between PGC-1α and NF-κB p65 based on Pearson correlation coefficients (mean ± SD). n=3, **p<0.01, ***p<0.001 (1-way ANOVA with Tukey post hoc test). Abbreviations: IP, immunoprecipitation; PGC-1α, peroxisome proliferatoractivated receptor γ; pNF, phosphorylated NF-κB; SIRT3, Sirtuin 3; TLR2, toll-like receptor 2.

PGC-1α is a major regulator of SIRT3 expression.^23^ Current evidence suggests that binding of PGC-1α with the p65 subunit of NF-κB can inhibit the transcriptional activity of PGC-1α, ultimately resulting in the downregulation of SIRT3 expression.^24^ Interestingly, a recent study implicated TLR2 as a key sensor recognizing HBV, which mediates response in hepatocytes by activating NF-κB-related signaling pathways in a MyD88-dependent manner.^25^ Inspired by these findings, we investigated whether HBV could regulate SIRT3 expression in macrophages through the TLR2-NF-κB-PGC-1α axis. Western blotting showed that treatment with HBV (10^7^ IU/mL) or Pam (TLR2 agonist^26^) could activate TLR2-NF-κB signaling in human THP-1 macrophages, which was validated by increased levels of phosphorylated NF-κB p65 and MyD88. Meanwhile, PGC-1α and SIRT3 expression was downregulated. Of note, the inhibitory effect of HBV on PGC-1α and SIRT3 expression was rescued following treatment with C29 (TLR2 inhibitor^27^) or BAY11-7085 (NF-κB activation inhibitor^28^), as shown in Figure 5A. Moreover, Co-IP assay results demonstrated that HBV stimulation led to increased binding of PGC-1α to the p65 subunit of NF-κB, similar to the effect of Pam, which was also suppressed by C29 or BAY11-7085 (Figure 5B).

Pearson correlation analysis based on immunofluorescence detection further validated the observation of Co-IP. Treatment with HBV or Pam promoted the colocalization of PGC-1α and p65 in human THP-1 macrophages. In contrast, inhibiting TLR2 with C29 prevented this increase in colocalized PGC-1α and NF-κB p65 (Figure 5C). Moreover, molecular docking analysis confirmed that PGC-1α could directly interact with NF-κB p65 (Supplemental Figure S5, http://links.lww.com/HC9/A587).

In summary, these results suggest that HBV-mediated aberrant activation of the TLR2-NF-κB-PGC-1α axis in macrophages significantly contributed to the downregulation of SIRT3 expression.

### Activating the TCA cycle can prevent M2-like polarization in the chronic HBV-infected mouse model

**FIGURE 6:**
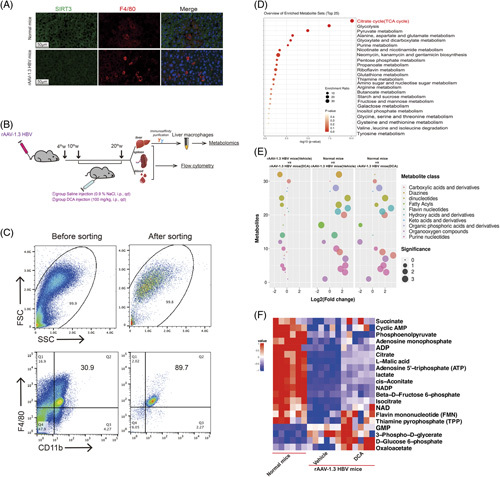
DCA treatment improves the TCA cycle flux of macrophages in the chronic HBV-infected mouse model. (A) SIRT3 expression of macrophages in liver sections from mice groups. F4/80 staining was used to label macrophages, and nuclei were stained with DAPI (blue) (magnification: 63×, scale bar: 50 μm). (B) Flowchart for DCA treatment of rAAV-1.3 HBV mice. The endpoint was set at 10 weeks from treatment initiation. (C) Isolated liver macrophages analyzed by flow cytometry to determine sorting efficiency and purity. (D) Enriched metabolic pathways related in liver macrophages from normal mice, vehicle-treated, or DCA-treated rAAV-1.3 HBV mice. (E) Targeted metabolomics data for liver macrophages from different groups. Metabolite classes are represented with different colors. Circle sizes reflect the significance of the log2 fold change. (F) Metabolomics data visualized by heatmap. Data presented as the mean ± SD, n=5. Abbreviations: DCA, dichloroacetate; FMN, flavin mononucleotide; FSC, forward scatter; GMP, guanosine monophosphate; rAAV, recombinant adeno-associated virus; SIRT3, Sirtuin 3; SSC, side scatter; TCA, tricarboxylic acid; TPP, thiamin pyrophosphate.

To establish an animal model that mimics chronic HBV infection in humans, we injected healthy C57BL/6 mice with rAAV-1.3 HBV by means of the tail vein. It is widely acknowledged that this model exhibits the characteristics of persistent viremia and anti-HBV immune response deficiency, effectively reflecting the immune tolerance mediated by HBV.^18,29,30^ Immunofluorescence assays revealed that the SIRT3 expression of liver macrophages in rAAV-1.3 HBV mice was significantly lower than in normal mice (Figure 6A), consistent with the *in vitro* findings and suggests impairment of the TCA cycle in macrophages (Figure 3A). Six weeks after rAAV-1.3 HBV injection, mice with comparable serum levels of HBsAg, HBeAg, and DNA copies were randomly divided into 2 groups. One group received DCA (an agonist of the TCA cycle^31,32^) treatment (100 mg/kg, i.p., once a day^19^), while the other received a saline (vehicle) injection (0.9% NaCl, i.p., once a day). Ten weeks after DCA treatment, the liver, spleen, and peripheral blood were harvested from each mouse for subsequent analysis (Figure 6B).

Liver macrophages were isolated from liver tissues (~purity: 89.7% assessed by flow cytometry; Figure 6C). Metabolomic analysis showed that the enriched metabolite sets of the liver macrophages from different mice groups (normal mice, vehicle-treated or DCA-treated rAAV-1.3 HBV mice) were associated with the TCA cycle, glycolysis/gluconeogenesis, and pyruvate pathway in glucose metabolism (Figure 6D). Meanwhile, other metabolite classes, namely, diazines, organic phosphoric acids and their derivatives, as well as keto acids and their derivatives, exhibited significant differences between groups (Figure 6E). Heatmaps were generated, revealing that the TCA cycle-related metabolites, including cis-aconitate, citrate, isocitrate, ATP, and NAD, were significantly downregulated in liver macrophages from vehicle-treated rAAV-1.3 HBV mice compared with those from normal mice; however, the downregulation in TCA cycle-related metabolites was alleviated in DCA-treated rAAV-1.3 HBV mice (Figure 6F). The observed changes in other metabolites associated with glucose metabolism are shown in Supplemental Figure S6, http://links.lww.com/HC9/A588. Taken together, these findings indicate that DCA treatment could partially rescue the impaired TCA cycle of macrophages in rAAV-1.3 HBV mice.

**FIGURE 7:**
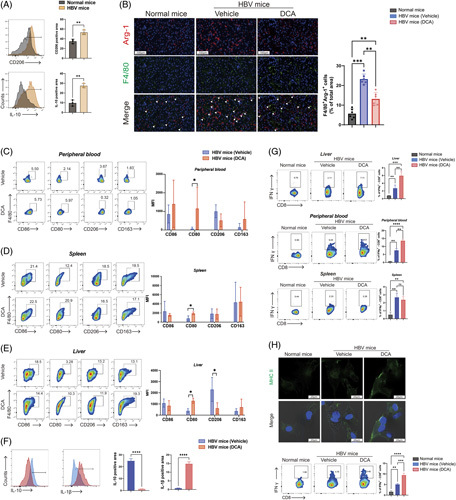
DCA treatment enhances the immune responses of macrophages in the chronic HBV-infected mouse model. (A) The expression of IL-10 and CD206 of liver macrophages from normal mice and rAAV-1.3 HBV mice were assessed by flow cytometry. Data presented as the mean ± SD, n=3, **p<0.01 (Student *t* test). (B) Immunofluorescence staining of Arg-1 (red) and F4/80 (green) in liver sections from mice. Nuclei were stained with DAPI (blue). Representative images shown in the left (magnification: 200×, scale bar: 100 μm). White arrows indicated doubly stained cells. Statistical analysis showed the ratio of double-stained cells in the right panel. (C–E) Expression of CD80, CD86, CD163, and CD206 in macrophages from mice livers, spleens, and peripheral blood. Data presented as the mean ± SD, n=5, ns=*p*>0.05, *p<0.05 (Student *t* test). (F) Expression of IL-10 and IL-1β in macrophages from mice livers. Data presented as the mean ± SD, n=3, ****p<0.001 (Student *t* test). (G) The frequencies of CD8^+^ IFN-γ^+^ T cells in liver, peripheral blood, and spleen of rAAV-1.3 HBV mice after DCA treatment. Data presented as the mean ±SD, n=6, ns=*p*>0.05, *p<0.05, **p<0.01, ***p<0.001 (1-way ANOVA with Tukey post hoc test). (H) Upper: Representative immunofluorescence images presenting the expression of MHCII on macrophages (green: MHCII, blue: DAPI) (magnification: 63×, scale bar: 20 μm). Lower: The frequencies of IFN-γ^+^ HBV-specific CD8^+^ T cells of different groups. Data presented as the mean ± SD, n=3, *p<0.05, **p<0.01, ***p<0.001, ****p<0.001 (1-way ANOVA with Tukey post hoc test). Abbreviations: DCA, dichloroacetate; MFI, median fluorescence intensity; MHCII, major histocompatibility complex class II; rAAV, recombinant adeno-associated virus.

Next, we studied the effect of activating the TCA cycle on the immune response of macrophages in rAAV-1.3 HBV mice. We first sought to verify the liver macrophage immunosuppressive state. Flow cytometry was used in order to quantify the expression of CD206 and IL-10 in liver macrophages, respectively. The results depicted in Figure 7A demonstrated that the liver macrophages from rAAV-1.3 HBV mice (orange peak) gave a significantly promoted signal intensity relative to that of normal mice (dark gray peak). Next, immunofluorescence staining with anti-F4/80 and anti-Arg-1 revealed that the expression of the Arg-1 in macrophages, which reflect immunosuppression in the liver microenvironment, was increased in liver tissues from rAAV-1.3 HBV mice compared to normal mice (Figure 7B, left). The statistical graph is presented in Figure 7B (right). We further examined the expression of markers (CD86 and CD80) associated with the immune-activated macrophage phenotype (M1) and the M2 phenotype (CD206 and CD163) in macrophages from rAAV-1.3 HBV mice (with or without DCA treatment) livers, spleens, and peripheral blood by means of flow cytometry. Following DCA treatment, CD80 expression increased in macrophages from the livers, spleens, and peripheral blood, while CD206 decreased in macrophages from liver tissues (Figure 7C–E). In particular, liver macrophages of DCA-treated mice secreted more IL-1β and less IL-10 than vehicle-treated mice (Figure 7F). It has been established that M1 macrophages contribute to the activation of T cells.^33^ Correspondingly, we found enhanced T-cell responses in DCA-treated rAAV-1.3 HBV mice with a significantly increased relative frequency of CD8^+^ IFN-γ^+^ T cells in liver and peripheral blood, but no statistical difference in spleen (Figure 7G). To validate the effect of DCA-treated macrophages on CD8^+^ T cells, the liver macrophages were firstly isolated and cultured with DCA and HBV (or without HBV as CON group) for 48 hours, then cocultured with CD8^+^T cells for another 6 hours. The expression of stimulatory marker major histocompatibility complex class II on the macrophages and the frequencies of CD8^+^ IFN-γ^+^ T cells are analyzed in Figure 7H. The results showed that expression of major histocompatibility complex class II by macrophages was elevated after HBV stimulation, a further increase under DCA administration (Figure 7H, upper). Meanwhile, CD8^+^ IFN-γ^+^ T cells increased when cocultured with HBV-induced macrophages, while the proportion of IFN-γ^+^ T cells was further augmented under DCA administration (Figure 7H, lower panel). In summary, these findings suggest that the activation of the TCA cycle *in vivo* plays a role in preventing the HBV particles from inducing M2-like polarization of macrophages. As a result, this activation triggers the innate and adaptive immune responses of the host to combat HBV infection.

Finally, we used PBMCs collected from patients with chronic HBV infection whose characteristic was showed in Supplemental Table S1, http://links.lww.com/HC9/A642 to further validate the effect of DCA treatment. Following treatment with DCA for 48 hours, we observed higher expression of M1 markers (CD86 and CD80) in PBMC-derived macrophages; however, this result was not statistically significant (Supplemental Figure S7, http://links.lww.com/HC9/A589), which may be due to the small sample size and individual variation.

### Activating the TCA cycle promotes HBV clearance in the chronic HBV-infected mouse model

**FIGURE 8:**
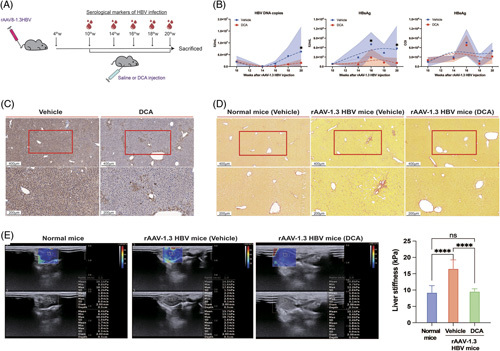
DCA facilitates HBV clearance and delays the development of liver fibrosis in the chronic HBV-infected mouse model. (A) HBeAg, HBsAg, and HBV DNA detection in murine peripheral blood samples collected at different time points during DCA treatment. (B) Serological markers of HBV infection (HBeAg, HBsAg, and HBV DNA) detected to assess HBV clearance. Data presented as the mean ± SD, n=8, ns=*p*>0.05, *p<0.05 (2-way ANOVA with Tukey post hoc test). (C) Immunohistochemistry for HBsAg in liver sections from mice. Representative images presented in the upper panel are magnified views of corresponding images in the bottom panel (magnification: 50×/100×, scale bar: 400 μm/200 μm). (D) Collagen deposition in liver tissues (magnification: 100×, scale bar: 200 μm; 50×, 400 μm). (E) Liver stiffness assessment based on shear-wave elastography. Representative shear-wave elastography images of mice are presented; the right bottom corner shows the corresponding statistical result for liver stiffness values obtained from shear-wave elastography. Data presented as the mean ± SD, n=6, ns=*p*>0.05, ****p<0.0001 (1-way ANOVA with Tukey post hoc test). Abbreviations: DCA, dichloroacetate; rAAV, recombinant adeno-associated virus.

Peripheral blood of rAAV-1.3 HBV mice was collected for HBeAg, HBsAg, and HBV DNA detection at weeks 1, 4, 6, 8, and 10 following the initial DCA injection (Figure 8A). From week 6, both serum HBsAg and HBV DNA levels were maintained at a lower level in rAAV-1.3 HBV mice that received DCA treatment compared with the vehicle-treated group, whereas the serum HBeAg levels were comparable between groups (Figure 8B). In addition, we assessed the tissue level of HBsAg in mice liver sections by immunohistochemistry, which was significantly decreased following DCA treatment compared with vehicle treatment (Figure 8C).

To determine whether DCA causes hepatotoxic effects, we compared ALT and AST levels between vehicle-treated and DCA-treated rAAV-1.3 HBV mice. At week 10 of DCA treatment, the ALT and AST levels of DCA-treated rAAV-1.3 HBV mice were 55.24±3.38 U/L and 20.87±1.24 U/L, respectively, and did not differ significantly from the vehicle-treated mice (ALT: 58.02±4.68 U/L, AST: 21.52±0.79 U/L). There was also no difference in the serum ALT and AST levels between pre-DCA treatment (ALT: 56.36±4.64 U/L, AST: 23.00±1.77 U/L) and post-DCA treatment (Supplemental Figure S8, http://links.lww.com/HC9/A590), indicating that DCA does not cause significant hepatotoxicity in rAAV-1.3 HBV mice.

Chronic HBV has been established as a major cause of liver fibrosis.^34^ Thus, the effect of DCA-induced activation of the TCA cycle on liver fibrosis development in rAAV-1.3 HBV mice was investigated. We performed Sirius red staining to determine the degree of liver fibrosis by observing collagen deposition. The liver tissues of vehicle-treated rAAV-1.3 HBV mice exhibited a stronger collagen deposition increase than normal mice (Figure 8D). Conversely, no significant evidence of fibrosis development was observed in the liver tissues from DCA-treated rAAV-1.3 HBV mice with patterns of collagen deposition comparable to those of normal mice. We used shear-wave elastography as an additional method to assess liver fibrosis. The liver stiffness value of vehicle-treated rAAV-1.3 HBV mice was significantly higher than normal mice (16.55±2.68 kPa vs. 9.23±2.11 kPa). In contrast, the liver stiffness of DCA-treated rAAV-1.3 HBV mice was 9.57±0.83 kPa, comparable with the normal mice (Figure 8E).

Collectively, these results suggest that inhibiting M2-like polarization by DCA-mediated TCA cycle activation could facilitate HBV clearance and delay the development of liver fibrosis.

## DISCUSSION

The chronic HBV infection process involves complex interactions between viruses and the host immune system. In this regard, macrophage-mediated innate immune responses constitute the first line of liver defense against viral infection. However, it has been reported that HBV can induce M2-like polarization, which inhibits innate immune responses and plays an important role in the pathogenesis of chronic persistent infection.^35–37^

Post-translational modifications have been shown to potently regulate innate immune responses by targeting innate sensors and downstream signaling molecules in response to pathogens.^38^ Here, we report that the acetylation levels of proteins, including CS and PDHC, are significantly increased in macrophages following exposure to HBV. The hyperacetylation of CS and PDHC can severely impact their enzymatic activities, contributing to the impaired TCA cycle. According to a recent study by Zhou et al^39^, diminished PDHC activity of macrophages could inhibit lipopolysaccharide-induced secretion of proinflammatory factors. The above studies overlap in their assertion that acetylation plays a critical role in coordinating cellular metabolism and immune responses in macrophages.

Next, we investigated how HBV infection induces hyperacetylation of CS and PDHC in macrophages. Pattern recognition receptors are key mediators for immune cells in sensing and responding to pathogens, while TLRs are a subfamily of pattern recognition receptors implicated in various inflammatory and infectious processes.^40,41^ It has been reported that exposure to low doses of lipopolysaccharide causes macrophage activation involving the TLR signal transduction pathway, which promotes inflammatory gene expression by means of induction of transcription factors, such as NF-κB and IFN regulatory factor. In contrast, long-term or high doses of lipopolysaccharide exposure can induce the feedback suppression of TLR signaling, causing macrophages to subsequently transform into immunosuppressive phenotype, a status of inflammation inhibition.^42,43^ However, the underlying molecular mechanism governing this phenomenon remains elusive. In the present study, we found that TLR2-NF-κB signaling was highly activated in macrophages during the M2-like polarization process mediated by HBV, with significantly enhanced binding of the NF-κB p65 subunit and PGC-1α. NF-κB P65 reportedly inhibits PGC-1α function and PGC-1α target gene transcription through protein-protein interactions, while SIRT3 functions as one of the downstream target genes of PGC-1α.^24^ As expected, the expression of SIRT3 (a strong deacetylase) and its interactions with CS and PDHC in macrophages were significantly weakened under HBV stimulation. By using a TLR2 agonist and inhibitor, we further confirmed that HBV could downregulate SIRT3 expression by means of the TLR2-NF-κB-PGC-1α axis, which accounts for the hyperacetylation of CS and PDHC in macrophages induced by HBV. In addition, enhancing the deacetylase activity of SIRT3 in macrophages restored HBV-mediated repression of CS and PDHC activities and prevented M2-like polarization. These results highlight the role of SIRT3 as a deacetylase for CS and PDHC during HBV-induced macrophage M2-like polarization. Interestingly, SIRT3 acts differently in different cells. It has been reported that as an NAD^+^-dependent protein deacetylase, SIRT3 is recruited to deacetylate cccDNA while blocking the recruitment of histone methyltransferase to cccDNA, ultimately inhibiting cccDNA transcription in the hepatocyte.^44^ Our findings suggest that SIRT3, which agonizes macrophages and hepatocytes, has a synergistic effect on eliminating HBV.

Activated macrophages commonly undergo glucose metabolism reprogramming to meet the bioenergetic needs of the macrophages during the immune response. In this respect, several metabolites from the TCA cycle are important regulators of macrophage functions during infection, autoimmunity, and cancer.^45^ For example, lipopolysaccharide promotes cytosolic citrate accumulation in macrophages by inducing the expression of the citrate carrier SLC25A1. Subsequently, under the action of ATP citrate lyase, cytosolic citrate is further converted into acetyl-CoA and oxaloacetate, which are necessary for the secretion of proinflammatory cytokines and the production of reactive oxygen species and nitric oxide.^46–48^ Despite these advancements, the feasibility of immune regulation by means of manipulating the TCA cycle in macrophages *in vivo* remains largely unexplored. Hence, we constructed a chronic HBV-infected mouse model (rAAV-1.3 HBV mice) to mimic the immunosuppression characteristic mediated by HBV. Next, we used DCA, an agent known to shift pyruvate metabolism from glycolysis and lactate production to glucose oxidation in the mitochondria,^32^ to improve the impaired TCA cycle of macrophages in rAAV-1.3 HBV mice. The results showed that DCA treatment decreased the abundance of M2-like macrophages in rAAV-1.3 HBV mice while increasing the abundance of CD8^+^ IFN-γ^+^ T cells, activating the innate and adaptive immune responses to some extent. By detecting the serological markers of HBV infection, we substantiated that activating the TCA cycle contributes to HBV clearance *in vivo*, and no significant development of liver fibrosis was observed in rAAV-1.3 HBV mice that received DCA treatment. The role of DCA in immune cell function and differentiation has recently been reevaluated, but it remains a subject of controversy. Some studies suggest that DCA can support humoral immunity in infectious disease models. On the other hand, DCA has also been found to induce regulatory T cells and suppress the differentiation of Th17 cells. These effects of DCA on T-cell differentiation are believed to be dependent on reactive oxygen species (ROS) production.^49–51^ Recent studies have reported that inhibition of succinate dehydrogenase (a vital enzyme in the TCA cycle) significantly impaired proliferation and neutralization of mitochondrial membrane potential and yielded a decrease in cytokine secretion like IFN-γ, which are all integral parts of T cell functionality.^52,53^ In addition, DCA suppressed macrophage migration by inhibiting glycolytic reprogramming.^54^ Moreover, DCA treatment had a major impact on ROS production in M1 and M2 macrophages.^55^ Specifically, succinate exerts its proinflammatory actions through many mechanisms, including stabilizing HIF-1α, affecting mitochondrial ROS production, and activating cell surface receptor, the G-protein–coupled receptor succinate receptor 1 in macrophages.^56^ Consistently, we found that promoting TCA cycling and increasing the levels of associated metabolites may play a role in enhancing the production of proinflammatory factors in macrophages, which can stimulate the secretion of cytokines by T cells. Benefiting from this, HBsAg and HBV DNA in the serum were decreased significantly after DCA treatment except HBeAg. Clinically, it is relatively uncommon that HBeAg levels did not drop with circulating HBV DNA on treatment, but it likely happens in rAAV-1.3 HBV mice.

Certain limitations found in our study must be acknowledged. First, although adeno-associated virus can reportedly introduce the HBV genome into the liver of immunocompetent mice and induce chronic HBV infection accompanied by persistent viremia for >30 weeks without the development of an anti-HBV immune response and spontaneous liver fibrosis,^18^ this model does not fully replicate the clinical presentation of chronic HBV infection. To overcome this limitation, we collected blood samples from patients with chronic HBV infection to verify the results observed in our animal study; however, future large-scale clinical validation studies are needed. Second, the present study focused on TCA cycle dysregulation in HBV-induced M2-like polarization. However, to the best of our knowledge, macrophage functions under pathological conditions are regulated by a complex interaction among multiple metabolic pathways. For example, in addition to glucose metabolism, the differential induction of fatty acid synthesis and oxidation drives tumor-associated macrophage polarization toward the M1 or M2 phenotype, respectively.^57,58^ Thus, future studies should explore the potential contributions of other metabolic pathways. Furthermore, our data shown that DCA administration acts on T cell to induce IFN-γ expression, which contributed to control HBV in vivo. So, the control of HBV infection in mice is probably the concerted action of multiple immune cells.

In summary, this study mainly reveals the interesting crosstalk between metabolic and immune processes in macrophages on HBV stimulation. In this respect, we demonstrated that HBV inhibits SIRT3 expression in macrophages by means of the TLR2-NF-κB-PGC-1α axis, resulting in hyperacetylation of CS/PDHC and impairment of the TCA cycle, contributing to M2-like polarization. Finally, we provide compelling evidence that exploiting the modulatory effect of the TCA cycle on the immune responses of macrophages may be an attractive strategy for controlling HBV infection.

## Supplementary Material
