# Supplementary material for: HBV suppresses macrophage immune responses by impairing the TCA cycle through the induction of CS/PDHC hyperacetylation
Source: Hepatol Commun. 2023 Oct 12;7(11):e0294. doi: 10.1097/HC9.0000000000000294 (PMC10578720; doi:10.1097/HC9.0000000000000294)
Supplement: Supplementary file 1 [file hc9-7-e0294-s001.pdf]

# HBV Suppresses Macrophage Immune Responses by Impairing the TCA Cycle through Induction of CS/PDHC Hyperactylation

First author: Jiaxin Bei[1*], Ye Chen[1*], Qianbing Zhang[2*]

## Supplementary table and figures

### Antibodies

The following antibodies were used throughout the study: anti-SIRT3 (sc-365175, Santa Cruz biotechnology, CA, USA), anti-acetyllysine (PTM-101, PTMBio, Hangzhou, China), anti-PGC-1α (A11971, Abclonal, Wuhan, China), anti-HBsAg (A30379, BOSTER, Wuhan, China), anti-F4/80 (70076S), and anti-CS (14309S, Cell Signaling Technology, MA, USA), anti-PDHC (18068-1-AP), anti-Arg-1 (16001-1-AP), and anti-β-actin (20536-1-AP, Proteintech, Wuhan, China), anti-TLR2 (WLH4120), anti-phospho-NF-κB p65 (WL02169), and anti-MyD88 (WL02494, Wanleibio, Shenyang, China), anti-NF-κB p65 (250060), Alexa Fluor 488 conjugated goat anti-mouse IgG (550036), Alexa Fluor 488 conjugated goat anti-rabbit IgG (550037), TRITC-conjugated goat anti-mouse IgG (511102), TRITC-conjugated goat anti-rabbit IgG (511202), HRP-conjugated goat anti-mouse IgG (511103), and HRP-conjugated goat anti-rabbit IgG (511203, ZENBIO, Chengdu, China).

The following antibodies were used for the flow cytometry analysis: PE-Cy7 anti-human CD163 (25-1639-42, Ebioscience, CA, USA), APC anti-human CD14 (555399), BB515 anti-human CD86 (564544), BV605 anti-human CD80 (563315)

and PE anti-human CD206 (555954, BD Biosciences, MA, USA), Alexa Fluor 700 anti-mice CD45 (103127), APC/Cy7 anti-mice CD11b (101226), PE/Cy7 anti-mice F4/80 (123114), PerCP/Cy 5.5 anti-mice CD86 (105028), APC anti-mice CD80 (104714), FITC anti-mice CD206 (141703), PE anti-mice CD163 (155308), Alexa Fluor 700 anti-mice CD45 (103127), BV510 anti-mice CD3 (100234), BV605 anti-mice CD8 (100744), BV650 anti-mice IFN γ (505832), PerCP/Cyanine5.5 anti-mouse IL-10 (505027, BioLegend, CA, USA), IL-1 beta (Pro-form) Antibody (17-7114-80, Thermo Fisher Scientific, MA, USA.), IL-1 beta Antibody (567779, BD Pharmingen, CA, USA) and Anti-Mouse MHC Class II (I-A/I-E) (65122-1-Ig, Proteintech, Wuhan, China).

**Cells**

The human monocytic cell line THP-1 (KeyGEN Biotech Co. Ltd) was cultured in RPMI-1640 with 10% FBS and 1% antibiotic-antimycotic supplementation (Complete Medium) at 37 °C and 5% $CO_2$. For HBV stimulation or drug treatment, THP-1 cells were differentiated into macrophages by incubating with 500 ng/mL phorbol myristate acetate for 3 h according to the previously described method [1]. According to different experimental purposes, the medium was subsequently replaced with fresh complete medium containing different titers of HBV ($10^3$, $10^5$, $10^7$ IU/mL), 100 ng/mL Pam3CSK4 (Pam; HY-P1180, MedChemExpress, NJ, USA), 50 μM C29 (HY-100461, MedChemExpress, NJ, USA) or 10 μM BAY11-7085 (HY-10257, MedChemExpress, NJ, USA); cells were continuously cultured for another 48 h.

Macrophages were cultured with fresh Complete Medium, then added in HBV ($10^7$ IU/mL) and nicotinamide riboside chloride (NR, 500 μM) (S2935, Selleck, TX, USA) simultaneously. Cells were continuously cultured for 48 hours, then harvested and used for flow cytometry experiments, co-localization analysis, Co-immunoprecipitation, Western blotting, enzymatic activity or cytokine detection assay, respectively.

Peripheral blood mononuclear cells (PBMCs) were purified from human or mice peripheral blood by density gradient centrifugation using Ficoll-Paque (17-1440-04, GE Healthcare, WI, USA) [2]. Using CD14 magnetic beads (130-050-201, Miltenyi) to isolate moncytes from Peripheral blood mononuclear cells. After obtaining moncytes, RPMI 1640 complete medium containing 50 ng/mL M-CSF and 20 ng/mL IL-4 was used to culture cells for another three days until it differentiated into macrophages. Human peripheral blood samples were collected from sixteen eligible individuals from the Second Affiliated Hospital, Guangzhou Medical University. Eligibility criteria included: (1) Age ≤ 30; (2) Normal liver function; (3) HBV DNA ≥ 4000 IU/mL; (4) HBsAg-positive; (5) HBeAg-positive; (6) CHB infection ≥ 6 months and without any antiviral therapy. All individuals provided signed informed consent, and the study was approved by the Clinical Research Review Committee of the Second Affiliated Hospital of Guangzhou Medical University (Approval No: 2021-YJS-ks-06). The clinical information regarding enrolled patients is listed in Supplementary information, Table S1.

Purified PBMCs were maintained in RPMI-1640 containing 10% FBS and 1%

antibiotic-antimycotic supplementation. HBV stimulation or NR treatment was processed by the same method as that described for THP-1 cells. For dichloroacetate (DCA) treatment, the culture medium was removed and replaced with complete medium containing 100 μM DCA; the cells were further incubated at 37 °C for 48 h.

Murine primary liver macrophages were isolated as previously described [3]. Briefly, a mouse liver was perfused via the portal vein with 0.05% collagenase in Hank's balanced salt solution (88284, Thermo Fisher, MA, USA). Vascular clamps were applied to stop the arterial and venous blood flow when the liver was filled with collagenase solution. Next, the liver was removed and digested at 42 °C for 10 min. The obtained suspension was resuspended in DMEM containing 5% FBS. Liver macrophages were purified from the sample via flow cytometric sorting using PE/Cy7 anti-mice F4/80 and APC/Cy7 anti-mice CD11b antibodies.

To examine T cell activation by macrophages treated with DCA, liver macrophages were isolated from HBV mice and then treated with or without DCA for 48h. T cells from spleen were directly co-cultured with macrophages above for another 6h. immunofluorescence staining were performed for macrophages (Anti-Mouse MHC Class II (I-A/I-E), 65122-1-Ig, Proteintech, Wuhan, China) and flow cytometry were performed for T cells.

**Targeted metabolomics analysis**

The targeted metabolomics analysis was performed by the Shanghai Applied Protein Technology Company (Shanghai, China). For metabolite extraction, cells

were collected and washed; the extract solution (acetonitrile: methanol: water = 2:2:1) was then added to each sample and vortexed for 60 s. Thereafter, the samples were kept in an ice-water bath and sonicated twice for 5 min each. Samples were stored at −20 °C for 1 h and centrifuged at 4 200 ×g for 20 min at 4 °C. The supernatant was collected and freeze-dried.

For liquid chromatography-mass spectrometry (LC-MS) analysis, HPLC separation was carried out using an Agilent 1290 series HPLC system (Agilent, CA, USA). The mobile phase consisted of 25 mmol/L ammonium acetate and 25 mmol/L ammonia hydroxide in water (pH = 9.75) (A) and acetonitrile (B). The elution gradient was as follows: 0-18.0 min, 90%-40% B; 18.0-18.1 min, 40%-90% B; 18.1-23 min, 90% B. The temperature of the column was maintained at 45 °C. The autosampler temperature was 4 °C, and the injection volume was 2 μL. The repeatability and stability of the system was evaluated by setting the quality control sample—prepared by mixing an equal aliquot of the supernatants from all samples—and injecting into the system every 6 samples throughout the sample cohort. The AB SCIEX QTRAP 5500 (Sciex Applied Biosystems, ON, Canada) was used to acquire mass spectrometry data in negative ion mode during the LC/MS experiment. The ESI source conditions were set as follows: source temperature, 450 °C; ion source Gas1 (Gas1), 45; ion source gas 2 (Gas2), 45; curtain gas (CUR), 30; ion Sapary Voltage Floating (ISVF)-4500 V. The multiple reaction monitoring mode was used for the measurement of ion pairs. Peak chromatographic area and retention time were analyzed with Multiquant software, and the metabolites were accurately identified by

correcting the retention times of each sample with those of known metabolite standards.

**Analysis of gene expression profiles**

Gene expression profiles of liver macrophages from mice with or without HBV infection were downloaded from the GEO database (accession: GSE165250) (https://www.ncbi.nlm.nih.gov/geo/). Differentially expressed genes between the different groups were identified using two-group comparisons. An adjusted $P < 0.01$ was set as the cut-off criterion.

**Tissue section staining**

Paraffin-embedded tissue sections were dewaxed, rehydrated and prepared for immunofluorescence, immunohistochemistry, and Sirius red staining, respectively.

*Immunofluorescence staining*: Antigen retrieval was performed by microwaving the sections in sodium citrate buffer (pH 6.0) for 15 min; endogenous peroxidase was blocked with 0.3% hydrogen peroxide in phosphate-buffered saline (PBS) for 5 min. After blocking with 10% goat serum, sections were incubated with primary antibodies against SIRT3 (1: 150), F4/80 (1: 125) or Arg-1 (1: 100) overnight at 4 °C. Alexa Fluor 488-conjugated goat anti-mouse antibody (1: 500) or TRITC-conjugated goat anti-rabbit antibody (1: 500) were subsequently added as secondary antibodies at room temperature for 1 h. Slides were mounted with DAPI-containing mounting medium (S2110, Solarbio, Beijing, China). The fluorescence images were observed

and recorded using an immunofluorescence microscope (ZEISS, Oberkochen, Germany).

*Immunohistochemistry staining*: Following antigen retrieval and endogenous peroxidase blockage, sections were incubated with primary antibodies against HBsAg (1: 200) overnight at 4 °C, followed by incubation with secondary HRP-conjugated goat anti-mouse IgG antibody (1: 500) for 1 h at room temperature. Color development was performed using the DAB chromogen kit (DA1016, Solarbio, Beijing, China), and counterstained with hematoxylin.

*Sirius red staining:* Sections were incubated with Sirius red staining solution (S8060, Solarbio, Beijing, China) for 8 min at room temperature and mounted with a neutral resin. The immunohistochemistry and Sirius red staining images were obtained using a DP70 digital microscope camera attached to a IX71 inverted microscope (Olympus, Tokyo, Japan).

**Confocal imaging and co-localization analysis**

Approximately 5 × $10^4$ THP-1 cells were harvested and fixed with 4% paraformaldehyde for 20 min at room temperature. Cells were then added to the slides and left to dry. Cells affixed to slides were permeabilized, blocked, and incubated with primary antibodies against SIRT3 (1: 150), CS (1:50), PDHC (1: 50), NF-κB p65 (1: 100), and PGC-1α (1: 100) overnight at 4 °C. Primary antibody-incubated cells were washed with PBS three times and incubated with Alexa Fluor 488 conjugated goat anti-mouse IgG antibody (1: 500) or TRITC-conjugated goat anti-rabbit IgG

antibody (1: 500) for 1 h at room temperature; nuclei were stained with DAPI. Co-localization of SIRT3 and CS/PDHC, as well as p65 and PGC-1α, was observed using a confocal microscope (LSM880) with a 63× oil objective (ZEISS, Oberkochen, Germany). For further quantification of fluorescent signal co-localization, the images were analyzed by calculating the Pearson correlation coefficient ($r$) with the ImageJ plugin Coloc 2 (https://imagej.net/plugins/coloc-*2*).

**Western blotting**

Western blotting was performed lysing cells with RIPA lysis buffer (P0013, Beyotime, Shanghai, China). Protein concentrations were determined using a Pierce™ BCA protein assay kit (Thermo Fisher, MA, USA). Equal amounts of protein were then separated using 10% sodium dodecyl sulfate-polyacrylamide gel electrophoresis (SDS-PAGE; 10%) and transferred to polyvinylidene difluoride (PVDF) membranes (Millipore, MA, USA). The membranes were blocked with TBS-Tween (TBST) containing 5% non-fat milk powder and incubated overnight at 4 °C with the following primary antibodies: SIRT3 (1: 1 000), acetyllysine (1: 1 000), CS (1: 1 000), PDHC (1: 500), TLR2 (1: 2 000), NF-κB p65 (1: 1 000), phospho-NF-κB p65 (1: 1 000), MyD88 (1: 1 000), PGC-1α (1: 1 500), and β-actin (1: 5 000). After incubation for 1 h with anti-mouse or anti-rabbit IgG HRP-conjugated secondary antibodies (1: 2 000) at room temperature, protein bands were visualized by chemiluminescence using ECL reagents (1705061, Bio-Rad, CA, USA).

## Co-immunoprecipitation (Co-IP) and GST pull-down

For Co-IP assays, primary antibodies against acetyllysine (1: 50), SIRT3 (1: 100) or PGC-1α (1: 50) were diluted in IP buffer (120 mM NaCl, 20mM Tris-HCl [pH 8.0], 1mM EDTA, 0.5% NP40), respectively. Protein A/G-magnetic beads (L-2007, Biolinkedin, Shanghai, China) were added and incubated at 4 °C for 6 h with rotation to prepare bead-antibody complexes. The magnetic beads were then harvested via microcentrifugation and incubated with cell lysates at 4 °C for 6 h. The magnetic beads were isolated again, and protein was eluted using elution buffer (0.1 M glycine, pH 3.0). The pH of the eluted proteins was immediately neutralized by 1 M Tris-HCl (pH 8.0) and separated via SDS-PAGE gels and analyzed.

GST pull-down assays were performed using a GST-tag protein purification kit (IK-2004, Biolinkedin, Shanghai, China) according to the manufacturer's instruction. In brief, His-tag CS protein (RPB661Hu01, Cloud-Clone, TX, USA) or His-tag PDHC protein (RPH426Hu01, Cloud-Clone, TX, USA) was incubated with GST-tag SIRT3 protein (RPE913Hu01, Cloud-Clone, TX, USA) or GST control protein (HY-P70270, MedChemExpress, NJ, USA) in binding buffer containing glutathione-agarose beads at room temperature for 2 h with rotation. The bound protein was then wash and immunoblotted with the indicated antibody.

## RT-qPCR

Total RNA was extracted using the EasyPure® RNA Kit (ER101-01, TransGen Biotech, Beijing, China), and cDNA was synthesized using the TransScript®

All-in-One First-Strand cDNA Synthesis SuperMix (AT341-01, TransGen Biotech, Beijing, China). Subsequently, RT-qPCR was performed using the PerfectStart® Green qPCR SuperMix (AQ601-01, TransGen Biotech, Beijing, China) on a LightCycler 480 system (Roche, Mannheim, Germany). The PCR amplification program included an initial denaturation step at 94 °C for 30 s, followed by 40 amplification cycles of 94 °C (5 s), 50-60 °C (15 s), and 72 °C (10 s). Relative expression levels of the target mRNAs were determined using the Ct method, and *ACTB* was used as an internal reference gene. The PCR primer sequences used in this study are presented as below.

| **Primer name** | **Sequence** |
|---|---|
| Human *SIRT3* | 5′-ACCCAGTGGCATTCCAGAC-3′ (forward) |
| | 5′-GGCTTGGGGTTGTGAAAGAAG-3′ (reverse) |
| Human *ACTB* | 5′-ATTAAGGAGAAGCTGTGCTACGTC-3′ (forward) |
| | 5′-ATGATGGAGTTGAAGGTAGTTTCG-3′ (reverse) |
| Human *TGF-β1* | 5′-CTAATGGTGGAAACCCACAACG-3′ (forward) |
| | 5′-TATCGCCAGGAATTGTTGCTG-3′ (reverse) |
| Human *IL-10* | 5′-GACTTTAAGGGTTACCTGGGTTG-3′ (forward) |
| | 5′-TCACATGCGCCTTGATGTCTG-3′ (reverse) |
| Human *Arg-1* | 5'-GTGGAAACTTGCATGGACAAC-3' (forward) |

| | |
|---|---|
| | 5'-AATCCTGGCACATCGGGAATC-3' (reverse) |

**Detection of enzymatic activity**

The PDHC activity assay kit (BC0385) was purchased from Solarbio (Beijing, China), and the CS activity assay kit (MAK193) was purchased from Sigma Aldrich (Madrid, Spain). Whole cell lysates of THP-1 cells were harvested and prepared for the determination of PDHC and CS activities following the manufacturer's protocol. The enzyme activity was detected by a Synergy H1 hybrid multi-mode reader (BioTek, VT, USA) through colorimetric reaction. All results were normalized to the protein concentration of cell lysates.

**Enzyme-linked immunosorbent assay (ELISA)**

Human ELISA kits for detecting TNF-α (EHC103a), IL-1β (EHC002b), IL-10 (EHC009) and TGF-β1 (EHC107b) of THP-1 cells were purchased from NeoBioscience (Shenzhen, China), and used according to the manufacturer's instructions. The final results were normalized by total protein concentration.

**Flow cytometry**

Cell suspensions prepared from total spleens and livers, or freshly isolated PBMCs were resuspended in staining buffer (555028, BD Biosciences, MA, USA) and preincubated with Fc Receptor Blocking Solution (422301/156603, BioLegend, CA, USA) for 15 min at 4 °C. Human PBMCs were stained with APC anti-human CD14,

BB515 anti-human CD86, BV605 anti-human CD80, PE anti-human CD206 and PE-Cy7 anti-human CD163. Murine PBMCs were stained with Alexa Fluor 700 anti-mice CD45, APC/Cy7 anti-mice CD11b, PE/Cy7 anti-mice F4/80, PerCP/Cy 5.5 anti-mice CD86, APC anti-mice CD80, FITC anti-mice CD206, and PE anti-mice CD16. The proportion of $CD8^{+}$ cytotoxic T cells from mouse peripheral blood, liver and spleen tissues was determined using Alexa Fluor 700 anti-mice CD45, BV510 anti-mice CD3, BV605 anti-mice CD8 and BV650 anti-mice IFN γ. Dead cell exclusion was carried out using a Fixable Violet Dead Cell Stain Kit (L34964, Invitrogen, CA, USA). All data acquisition was performed with a BD LSRFortessa (BD Biosciences, MA, USA) and analyzed using FlowJo Software (FlowJo).

**Liver ultrasound shear-wave elastography (SWE) stiffness evaluation**

SWE assesses liver stiffness by measuring acoustically generated tissue shear-wave propagation speeds and has been regarded as an important indicator for the diagnosis of liver fibrosis [4]. Two-dimensional SWE was performed using a supersonic Aixplorer ultrasound system (Supersonic Imagine, Aixen Provence, France) with a 15-4 MHz linear transducer. Briefly, the mice were anesthetized with isoflurane and secured in the supine position during the scan. The abdominal skin was shaved before the ultrasound transmission gel was applied. A two-dimensional linear array probe was placed below the xiphoid process until the most adequate window for the liver was obtained. The SWE mode was activated in the selected liver area. The SWE image was presented in pseudocolor, in which each pixel with different values for stiffness

(kPa) was coded with a unique color. An appropriately sized region of interest was then established to include the liver tissue while avoiding adjacent vessels. The tissue elasticity properties were automatically obtained using self-contained software and included mean stiffness value (mean), minimum stiffness value (min), maximum stiffness value (max), and standard deviation (s.d.).

**Molecular docking**

The HDOCK software (*http://hdock.phys.hust.edu.cn/*) was applied to study the molecular docking of PGC 1α and NF-κB p65. The two co-crystal structures (PDB ID: 6KXY, 5URN) were obtained and applied to construct an energy grid. Default settings were employed for the grid generations and docking. Post-minimization was used to optimize the geometry of the poses.

## References:

**1.** Song H, Xu F, Pang X, et al. STAT3-Dependent Gene TRIM5gamma Interacts With HBx Through a Zinc Binding Site on the BBox Domain. *Front Microbiol.* 2021;12:663534.

**2.** Song JW, Zhang C, Fan X, et al. Immunological and inflammatory profiles in mild and severe cases of COVID-19. *Nat Commun.* 2020;11(1):3410.

**3.** Huai W, Liu X, Wang C, et al. KAT8 selectively inhibits antiviral immunity by acetylating IRF3. *J Exp Med.* 2019;216(4):772-785.

**4.** Ferraioli G, Tinelli C, Dal Bello B, Zicchetti M, Filice G, Filice C. Accuracy of real-time shear wave elastography for assessing liver fibrosis in chronic hepatitis C: a pilot study. *Hepatology.* 2012;56(6):2125-2133.
