# Supplementary figures and images for: HBV suppresses macrophage immune responses by impairing the TCA cycle through the induction of CS/PDHC hyperacetylation
Source: Hepatol Commun. 2023 Oct 12;7(11):e0294. doi: 10.1097/HC9.0000000000000294 (PMC10578720; doi:10.1097/HC9.0000000000000294)

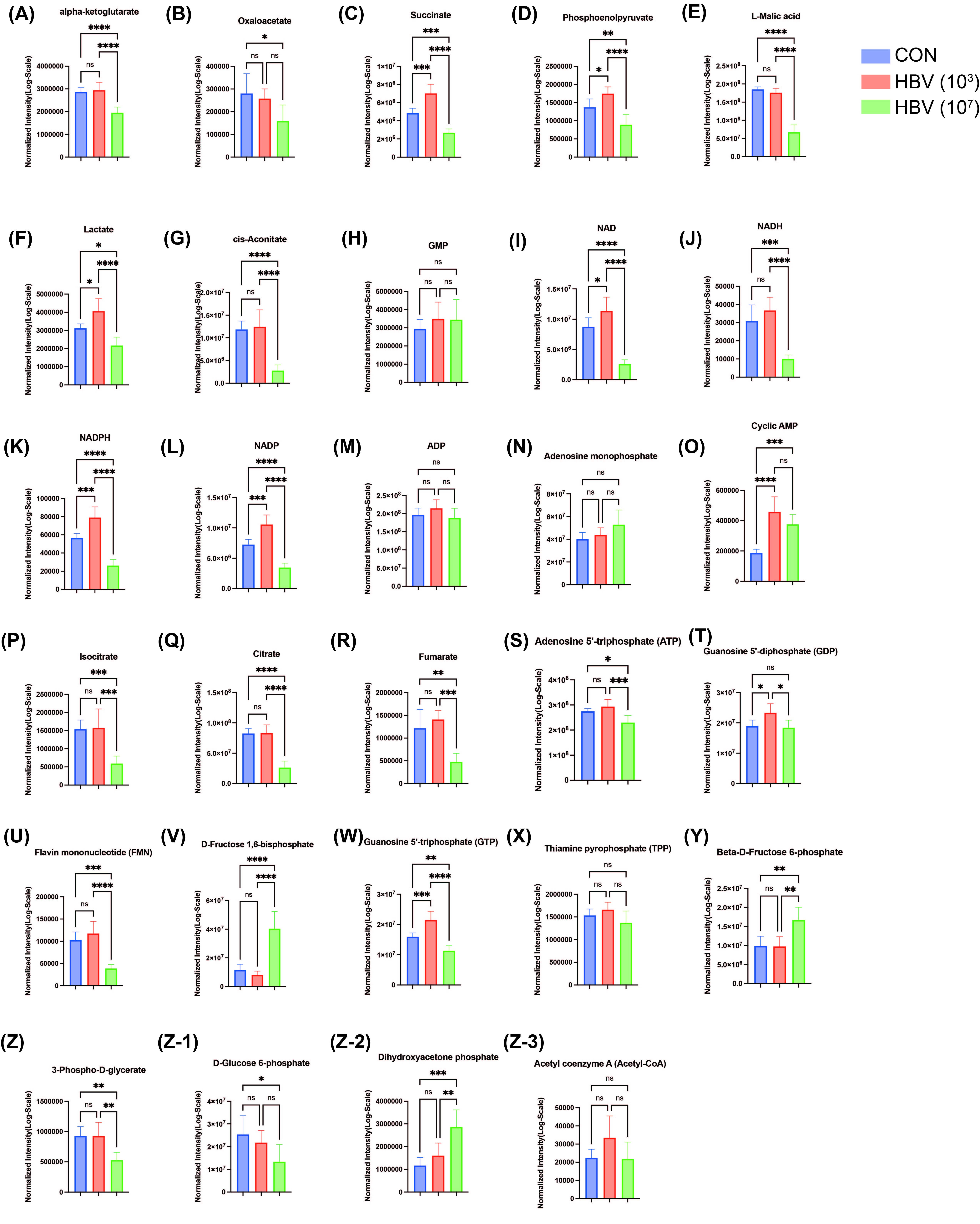

Supplement: Supplementary file 2 [file hc9-7-e0294-s002.jpg]

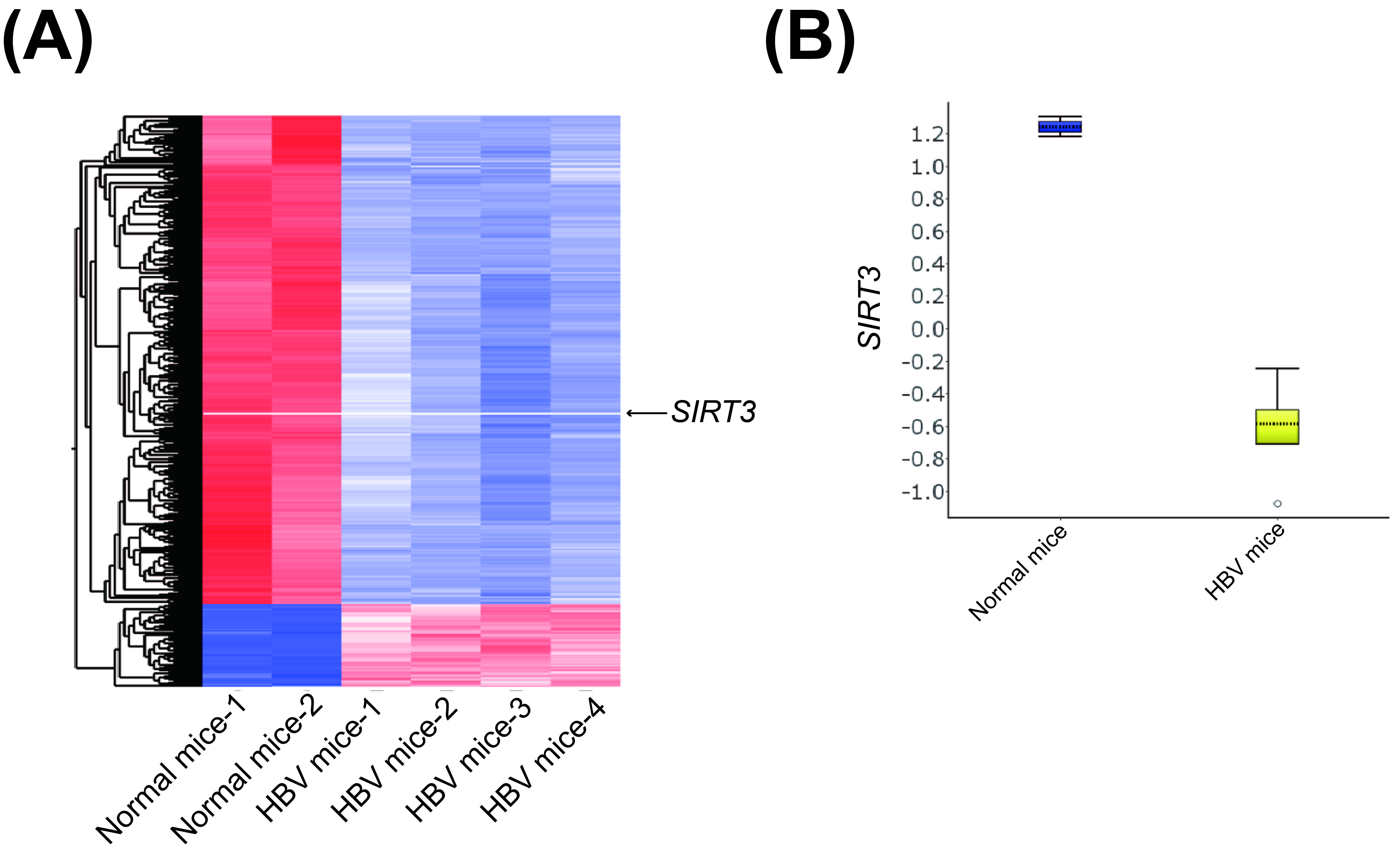

Supplement: Supplementary file 3 [file hc9-7-e0294-s003.jpg]

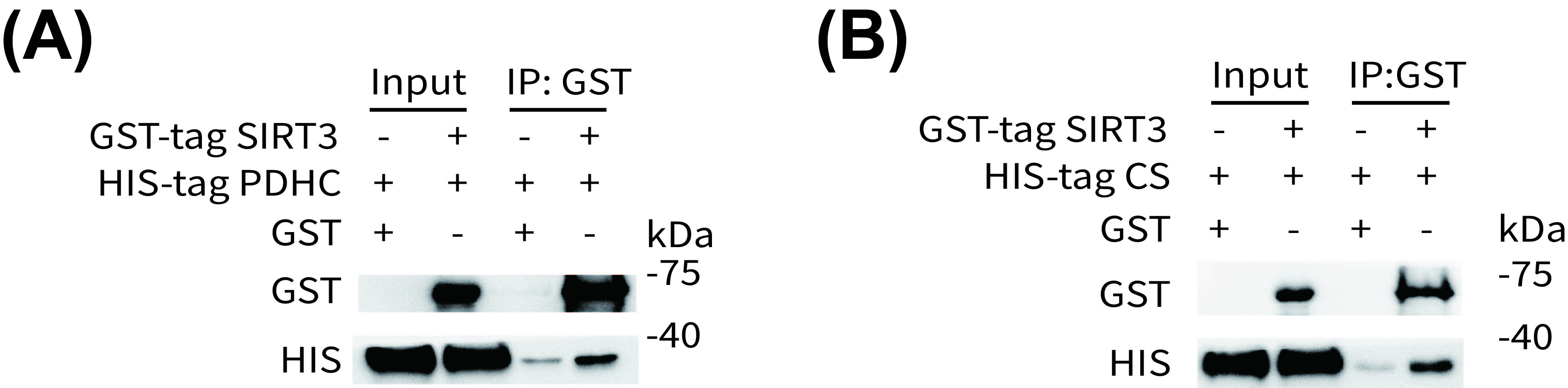

Supplement: Supplementary file 4 [file hc9-7-e0294-s004.jpg]

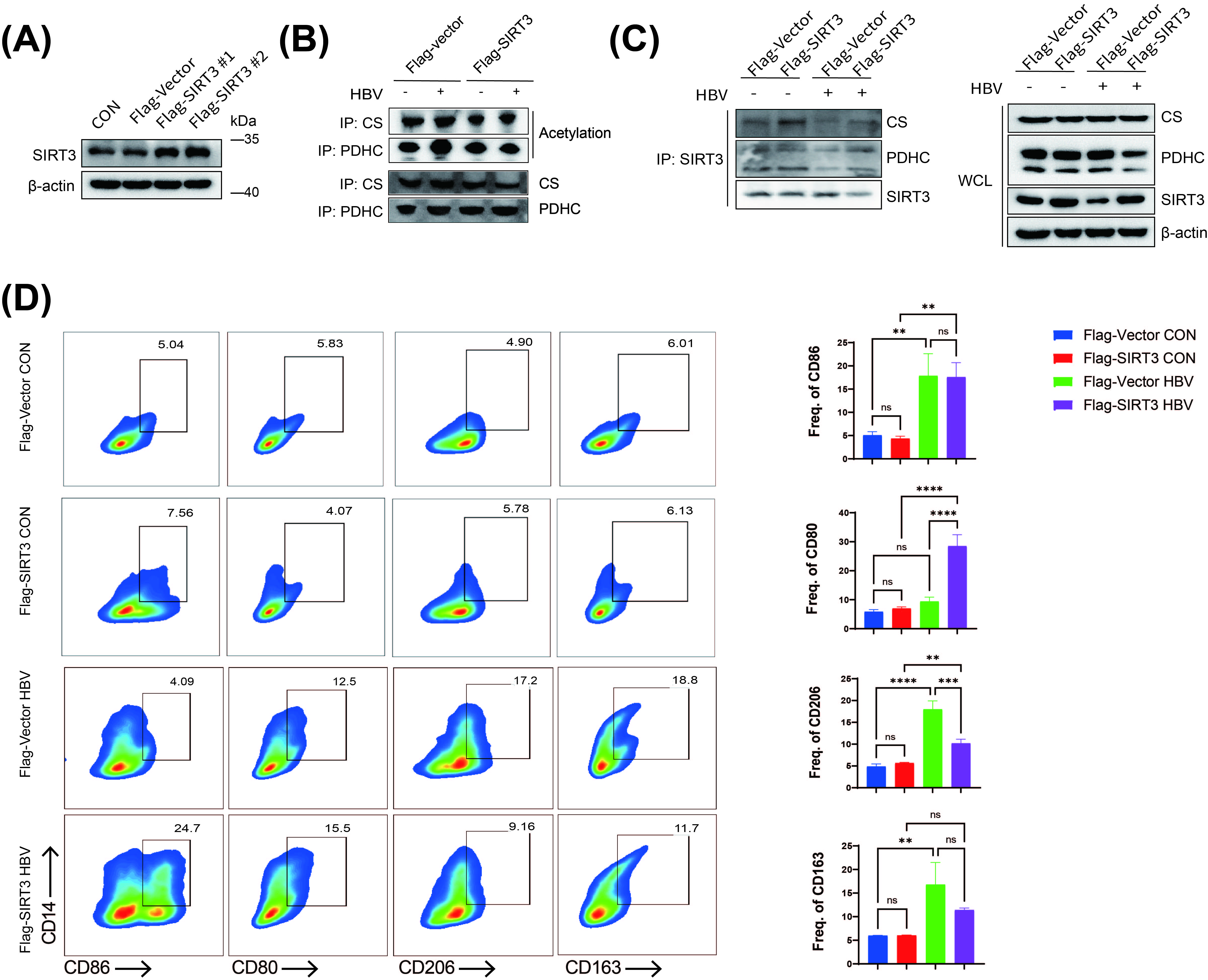

Supplement: Supplementary file 5 [file hc9-7-e0294-s005.jpg]

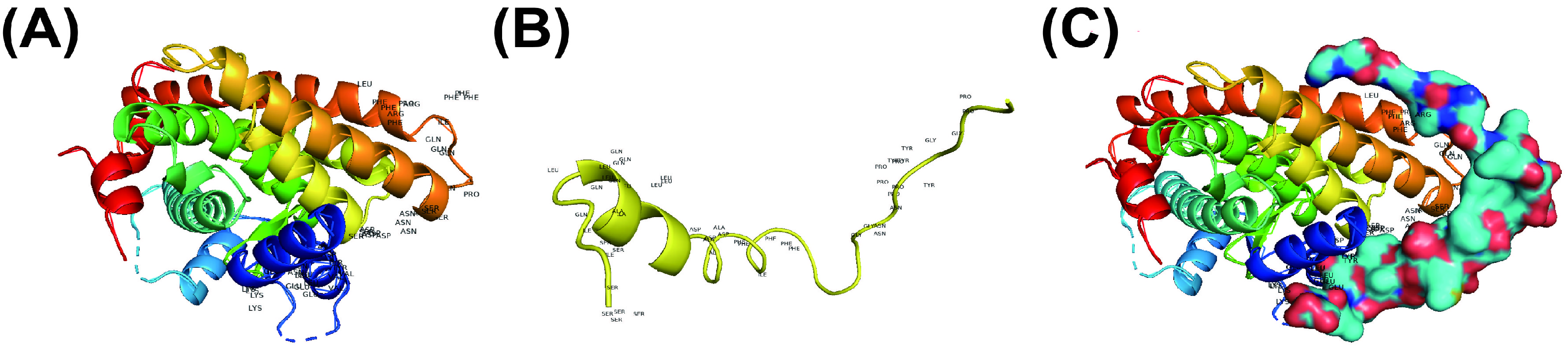

Supplement: Supplementary file 6 [file hc9-7-e0294-s006.jpg]

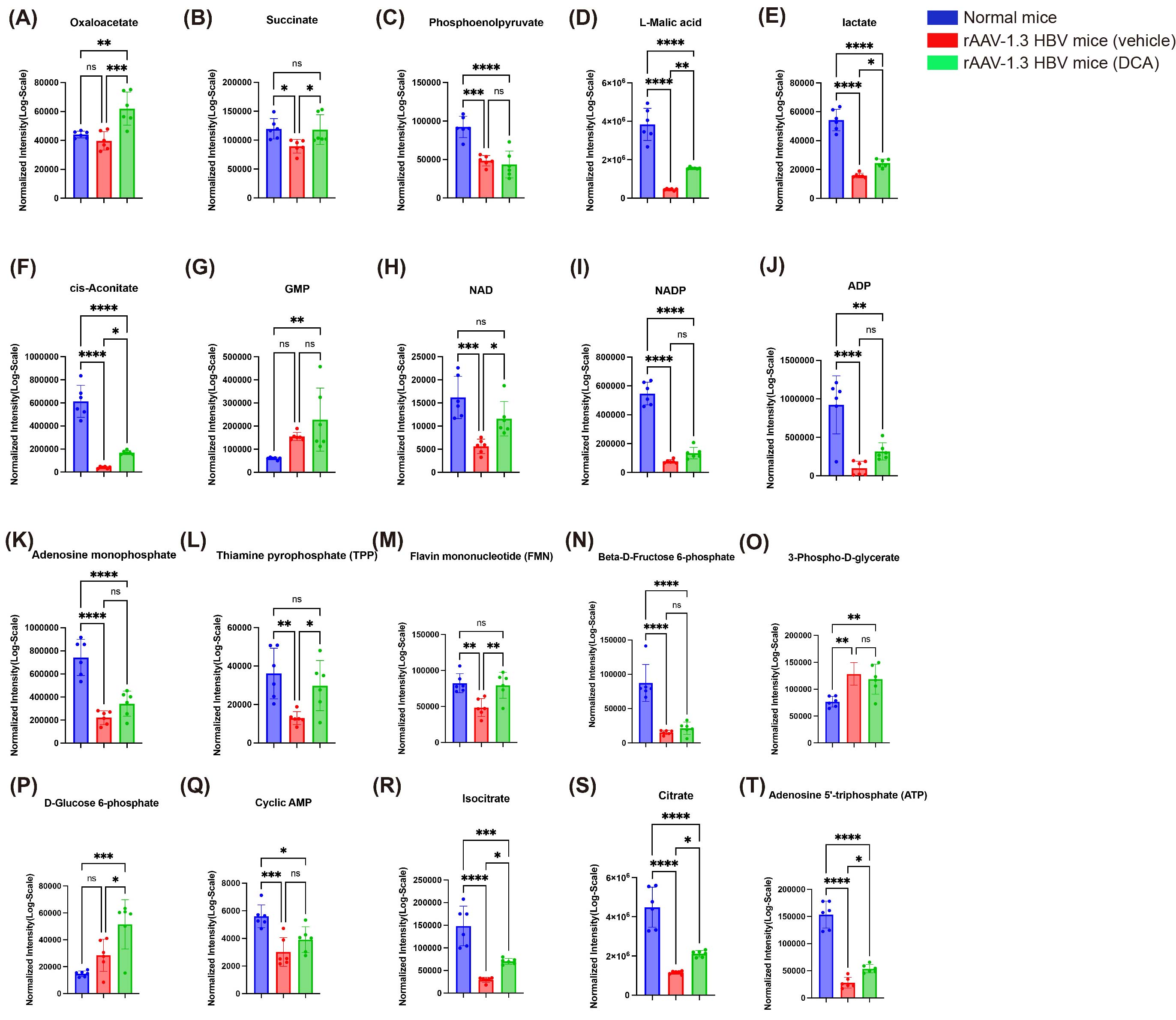

Supplement: Supplementary file 7 [file hc9-7-e0294-s007.jpg]

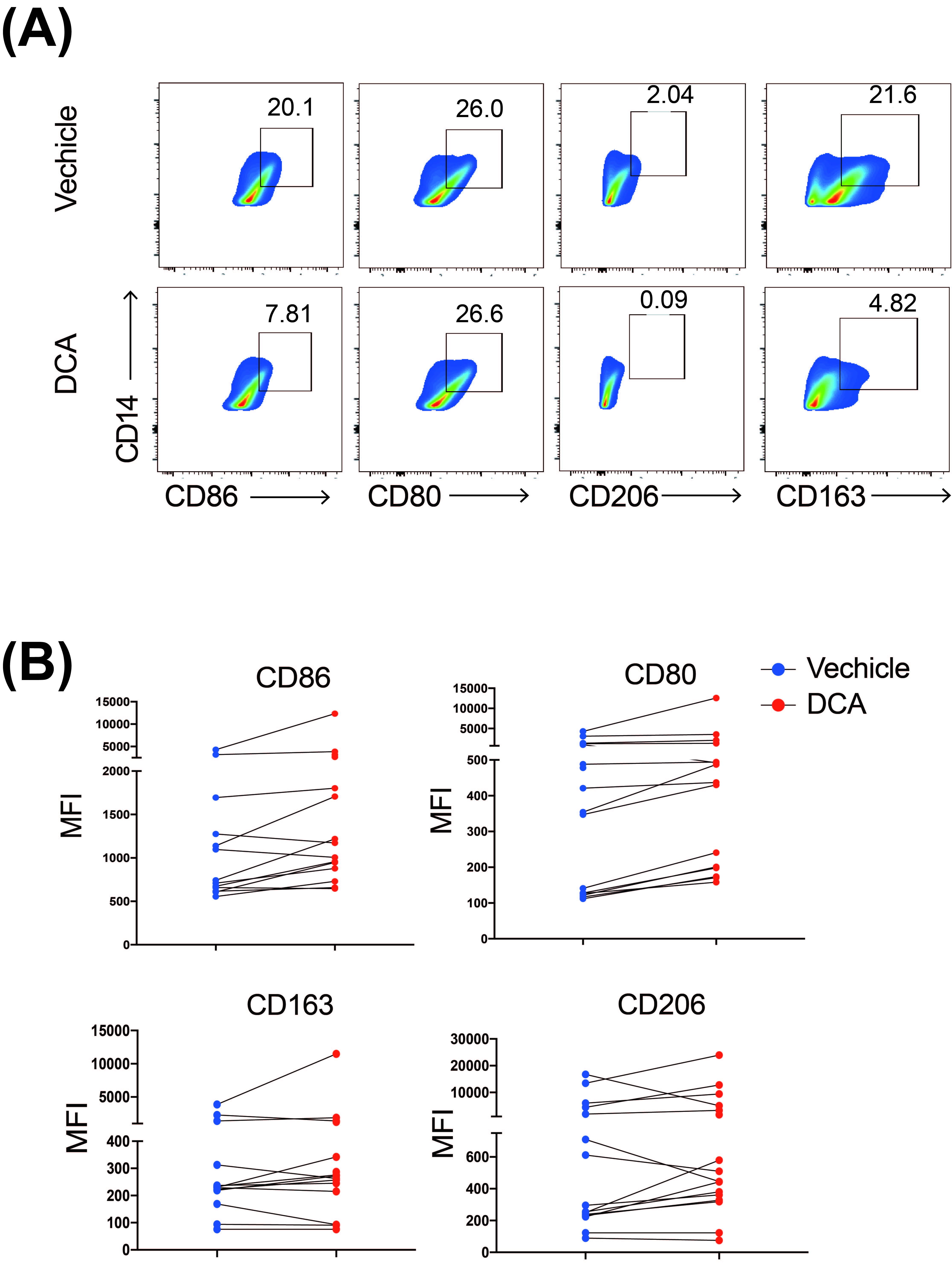

Supplement: Supplementary file 8 [file hc9-7-e0294-s008.jpg]

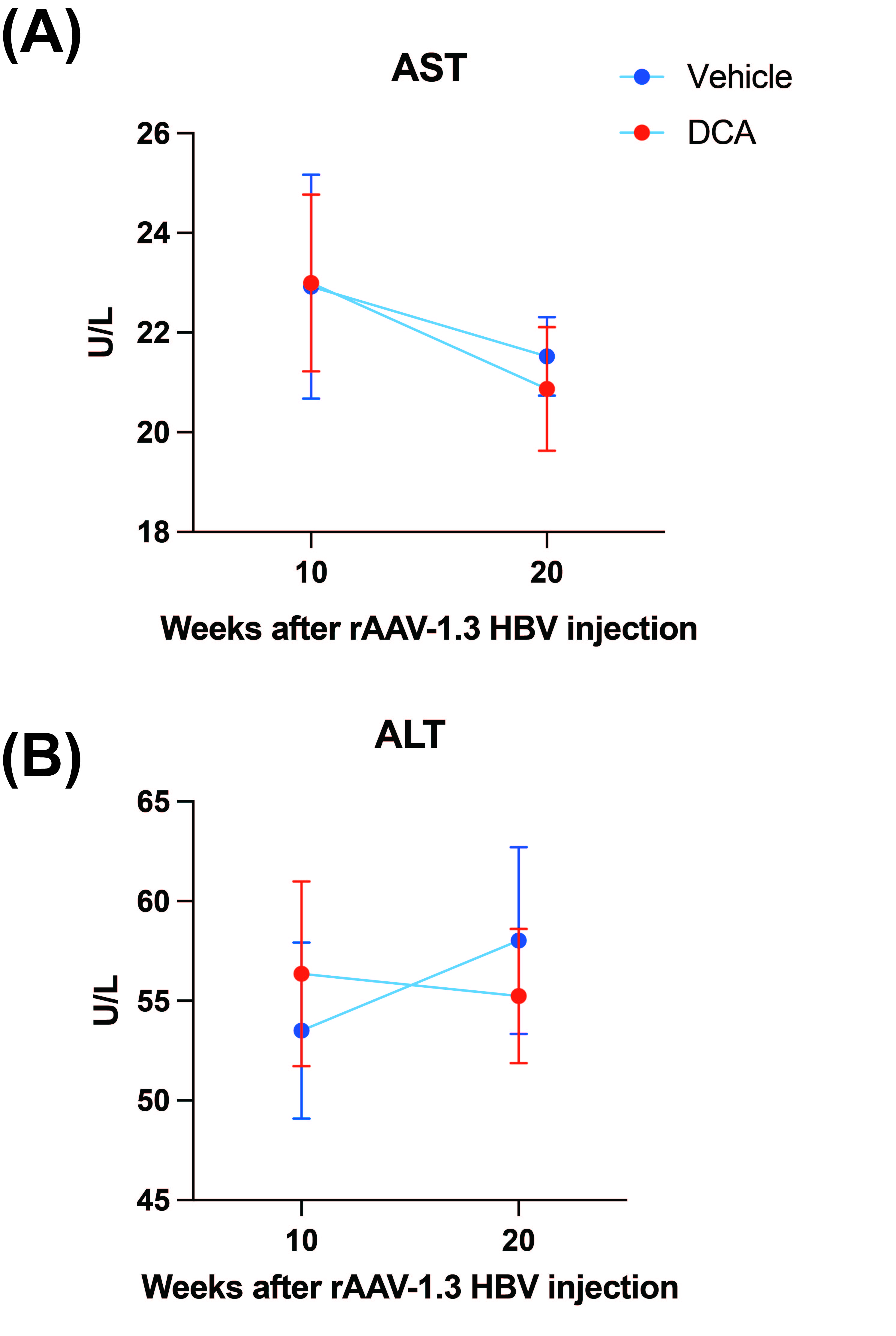

Supplement: Supplementary file 9 [file hc9-7-e0294-s009.jpg]
