# Supplementary material for: HBV suppresses macrophage immune responses by impairing the TCA cycle through the induction of CS/PDHC hyperacetylation
Source: Hepatol Commun. 2023 Oct 12;7(11):e0294. doi: 10.1097/HC9.0000000000000294 (PMC10578720; doi:10.1097/HC9.0000000000000294)
Supplement: Supplementary file 10 [file hc9-7-e0294-s010.pdf]

Table S1. Clinical characteristics of patients with chronic HBV infection.

**Clinical data**	
**Sex (male/female)**	7/9
**Age (y)**	27.9 ± 7.7
**HBV DNA (log10 IU/mL)**	5.9E+07 ± 8.4E+07
**HBsAg**	+
**HBeAg**	+
**ALT (U/L)**	37.5 ± 10.9
**AST (U/L)**	33.2 ± 8.6
**Accepted Anti-viral treatment**	0

HBV, hepatitis B virus; HBsAg, hepatitis B surface antigen; HBeAg, hepatitis B e antigen; ALT, alanine transaminase; AST, Aspartate transaminase. Data are shown as the mean ± s.d. (n = 16).
